# Acute Chagas Disease: New Global Challenges for an Old Neglected Disease

**DOI:** 10.1371/journal.pntd.0003010

**Published:** 2014-07-31

**Authors:** Daniela V. Andrade, Kenneth J. Gollob, Walderez O. Dutra

**Affiliations:** 1 Department of Morphology, Federal University of Minas Gerais, Belo Horizonte, Minas Gerais, Brazil; 2 National Institute for Science and Technology in Tropical Diseases, INCT-DT, Belo Horizonte, Minas Gerais, Brazil; 3 Hospital Santa Casa-BH, Institute for Education and Research, Graduate Program in Biomedicine and Medicine, Belo Horizonte, Minas Gerais, Brazil; Universidade Federal de Minas Gerais, Brazil

## Abstract

Chagas disease is caused by infection with the protozoan *Trypanosoma cruzi*, and although over 100 years have passed since the discovery of Chagas disease, it still presents an increasing problem for global public health. A plethora of information concerning the chronic phase of human Chagas disease, particularly the severe cardiac form, is available in the literature. However, information concerning events during the acute phase of the disease is scarce. In this review, we will discuss (1) the current status of acute Chagas disease cases globally, (2) the immunological findings related to the acute phase and their possible influence in disease outcome, and (3) reactivation of Chagas disease in immunocompromised individuals, a key point for transplantation and HIV infection management.

## Introduction

**Figure 1:**
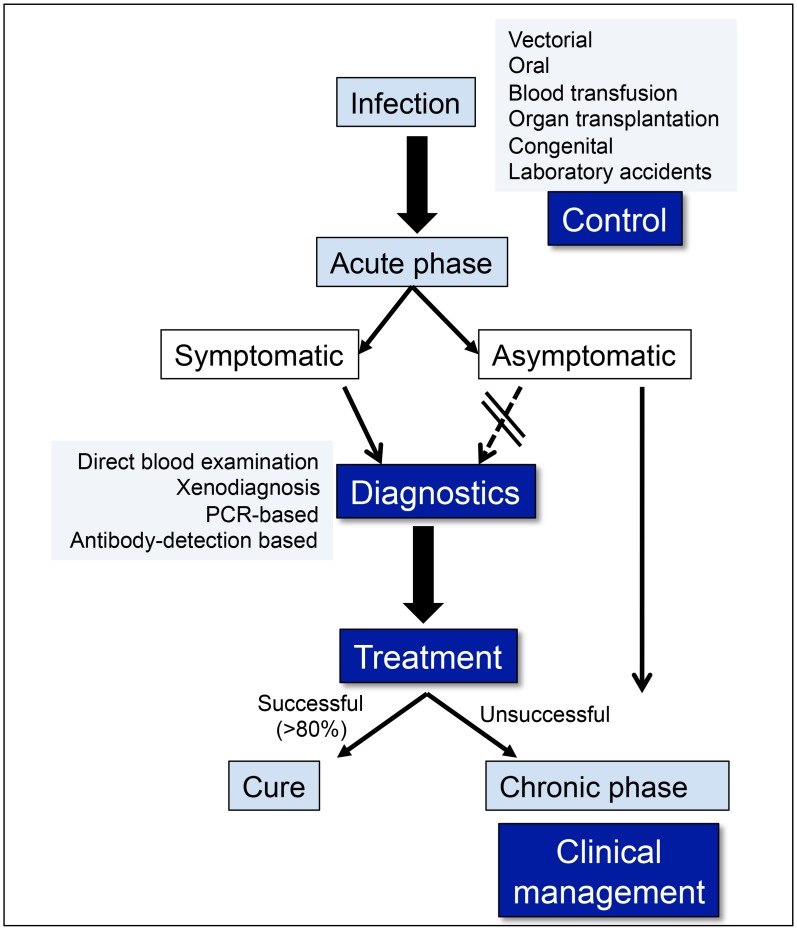
Challenges in human Chagas disease: control, diagnosis, treatment, and clinical management. Regardless of the route of infection, control of *T. cruzi* transmission is still a challenge, especially considering disease emergence and re-emergence, as discussed in this review. It is also critical to detect infection early on in order to provide immediate treatment to the patients. It is estimated that treatment efficacy is observed in at least of 80% of treated acute patients. Lack of detection of the acute phase or treatment failure lead to disease chronification. Given that approximately 30% of the patients in the chronic phase will develop severe, clinical forms of Chagas disease, which often lead to death, clinical management is critical. However, given that the mechanisms responsible for patient progression from the indeterminate to the symptomatic forms of Chagas disease are not completely understood, clinical management presents another important challenge. The search of prognostic markers of disease progression is a critical aspect for preventing pathology and introducing better clinical measures.

In 2010, the 63rd World Health Assembly passed resolution WHA63.20, highlighting the seriousness of Chagas disease in both endemic and non-endemic countries, which called for measures to address Chagas disease transmission, diagnostics, and treatment at all levels. The increasing presence of Chagas in non-endemic areas (mostly due to immigration, blood transfusion, and organ transplantation), as well as the resurgence of the disease in endemic countries, has been a major focus of attention in recent years. The acute phase of Chagas disease is a critical period for this debilitating infection for many reasons: (1) it represents the first overt disease phase as a result of the host–pathogen interaction, often accompanied by serious symptoms, especially in patients infected by the oral route; (2) early detection in this phase allows for the introduction of effective and appropriate therapeutics; and (3) the immunological events that take place during the acute phase will likely influence disease outcome during the chronic phase, helping determine whether the patient will remain in the asymptomatic form, or progress to the deadly, cardiac, clinical form of the disease. These points highlight some of the greatest challenges in human Chagas disease, which are epidemiological control, efficient diagnosis and treatment, and clinical management (Figure 1).

## Chagas Disease Is Crossing Its Boundaries

Chagas disease is a vector-borne parasitic disease caused by the infection with the protozoan *Trypanosoma cruzi*. Chagas disease history has been associated with Latin America for over 9,000 years, where it is still endemic in many countries. However, it is currently estimated that 7 to 8 million people are infected by *T. cruzi* worldwide [1].

Several government-based control programs of Chagas disease transmission were launched in Latin America, starting as early as 1960. Amongst these programs was the Southern Cone Initiative (SCI), formalized in November 1991 by the governments of Argentina, Bolivia, Brazil, Chile, Paraguay, and Uruguay, with the main goal of containing disease transmission primarily by eliminating the principal domiciliary vector (*Triatoma infestans*) [2]. While great progress was achieved via these programs, they were not accompanied by exhaustive surveillance actions. As a result, areas that were previously considered vector-free are now repopulated with *T. cruzi*–infected vectors, leading to recent cases of acute Chagas disease.

**Figure 2:**
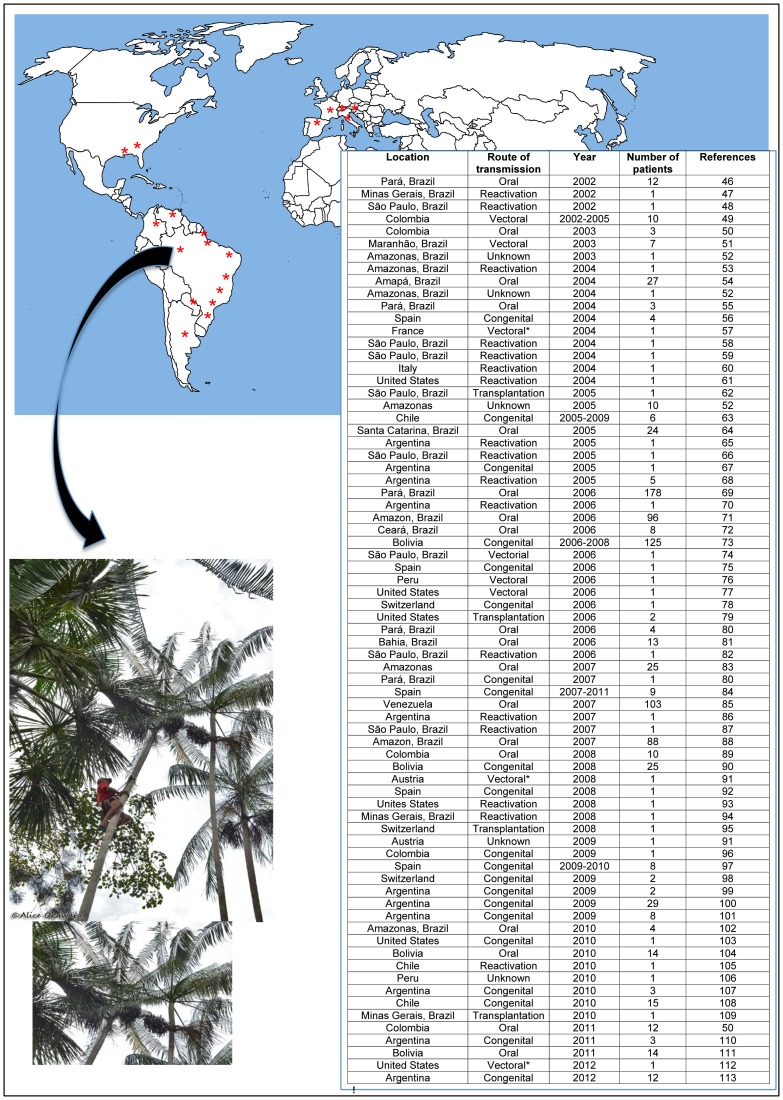
Acute cases of Chagas disease worldwide. The embedded table shows specific information on the transmission routes and number of affected individuals per case, when available. Asterisks (*) in the map indicate acute Chagas disease cases reported in the last ten years. Most cases in South America were due to vector transmission, congenital, and reactivation cases. In Brazil, the greatest number of cases were due to oral contamination. In Europe and the US, most cases were due to congenital or reactivation [46]–[113].

In the past ten years, an astonishing number of 73 reports of acute Chagas disease were found in the indexed literature (Figure 2), contrasting with 41 over the previous 20 years (1981–2001). Thus, the number of reported cases has at least doubled in the past ten years. The alarming numbers would be even greater, were it not for the underdiagnosed cases, both in endemic and non-endemic areas.

Oral infection with *T. cruzi* currently represents the most frequently documented route of transmission in Brazil. Micro epidemics of acute Chagas disease have been reported in the Amazon region and are mostly associated with consumption of contaminated açaí (*Euterpe oleracea*) palm fruit (usually found in the vicinities of the houses or nearby forest areas) (Figure 2) [3], as well as sugar cane juice [4]. It is challenging to determine with complete certainty if the human infections observed in these areas occur by the oral route, given that the presence of the vector may also lead to conventional transmission, but recent studies have implicated contaminated açaí consumption as one of the main causes for acute Chagas outbreaks in the Amazon region [3].

Increased travelling and migration of individuals from endemic to non-endemic areas has presented a worrisome scenario for congenital infection, blood transfusions, and organ transplantations. It has been estimated that over 300,000 people infected with *T. cruzi* currently live in the United States [5] and, although relatively limited, epidemiological data from Europe has estimated 59,000–108,000 cases of Chagas disease, with higher numbers in Spain and Italy [6]. Most documented cases refer to chronically infected individuals, but a few acute cases have also been identified in these regions (Figure 2). In view of the current situation, the US, France, Spain, and United Kingdom have instituted comprehensive blood bank and organ screening for *T. cruzi* [6].

Thus, a disease that was traditionally contained in Latin America, where it still predominantly occurs, has now crossed these boundaries. As it spreads, it becomes not only a problem of the endemic countries but also for the international community.

## Early Immunological Events Driving Disease Outcome: Evidence-Based Theories

The acute phase of Chagas disease represents the first contact between the parasite and the host, and the moment in which the immunological response will be triggered. It is possible (and likely) that the immunological events that take place during the acute phase will greatly influence the outcome of the disease towards the development of protective or pathogenic response in the chronic phase.

Detection of individuals during the acute phase of Chagas disease is rare because of the relatively nonspecific clinical symptoms observed in most of the infected patients [3], [4]. This delay in detection prevents diagnosis and consequently limits availability of studies describing the immunological status of acutely infected individuals. Moreover, late (or absent) diagnosis impairs treatment and, thus, disease cure. The use of conventional therapy, as well as the need for alternative drugs and/or adjuvants to adequately treat Chagas disease patients, are critical issues and were discussed by us in another review [7]. While scarce, the findings obtained so far have provided valuable information clarifying the role that initial immunological events might have on driving the development and progression of distinct clinical manifestations during the chronic phase of Chagas disease. Most immunological studies performed to date in acutely infected individuals followed the leads given by studies in experimental models, which are briefly summarized in Box 1.

**Box 1. Immunity in Acute *T. cruzi* Infection—Lessons from Murine Models**

During the early stages of infection, activation of macrophages and dendritic cells by pathogen-associated molecular patterns (PAMPs) will lead to the activation of these cells and subsequent production of IL-12. IL-12 induces IFN-gamma synthesis [33], which augments synthesis of IL-12 itself, TNF-alpha, and nitric oxide (NO) by macrophages, contributing to parasite clearance [34]. IFN-gamma also favours the recruitment of T cells by inducing expression of chemokines and adhesion molecules [35]. In the early phase of infection, most of the recruited mononuclear cells found in the focal inflammatory infiltrate surrounding *T. cruzi*–infected cardiomyocytes are CD8^+^ lymphocytes. Thus, during early stages of the experimental infection, the production of inflammatory cytokines and the activation of cytolytic cells may be critical for parasite control [36]. In fact, mice that are deficient in inflammatory cytokine production and in CD8+ T cell response are susceptible to *T. cruzi* infection [37]. CD4^+^ T cells play an important role, mostly as orchestrators of the immune response via cytokine production. Less frequent, yet functionally relevant T cell subpopulations, such as the CD4^−^CD8^−^ (double negative) and CD4+CD8+ (double positive) T cells, have been shown to participate in immunity to *T. cruzi*, although their function is still not completely clear [38], [39].

The ideal resolution of the acute phase should not only rely on controlling parasite dissemination, but also on down-regulating the immune system in order to avoid tissue damage. Increasing evidence supports the hypothesis that the fine balance between inflammatory and modulatory cytokines derived from distinct T cell sources is a key factor for preventing tissue damage to the host. The essential role of IL-10 in the immunomodulation was illustrated by experiments showing the role of this cytokine in balancing the TNF-alpha-driven pro-inflammatory response in *T. cruzi*–infected mice [40]. IL-17, which was shown in other conditions to have pro-inflammatory properties, controls cardiac inflammation by modulating Th1 response in mice acutely infected with *T. cruzi* [41].

The importance of antibodies for controlling *T. cruzi* infection was demonstrated by the transfer of sera from chronically infected mice to naive mice, which showed significantly reduced parasitemia after challenge with *T. cruzi* [42]. However, antibodies are not only involved in the resistance to *T. cruzi*, but may also mediate tissue destruction. An important feature of this response is the preferential activation of CD5^+^ B cells [43], which had already been closely related to autoimmune disorders.

The acute infiltration of immune cells in *T. cruzi*–associated myocarditis is induced by a Th1-biased immune response, with high expression of the inflammatory cytokines IL-6, IL-1, and TNF-alpha [44], [45], which control the expression of chemokines and adhesion molecules. While this inflammatory reaction controls parasite replication and parasitism, it is also the main cause of tissue injury, leading to morbidity and mortality [36].

**Figure 4:**
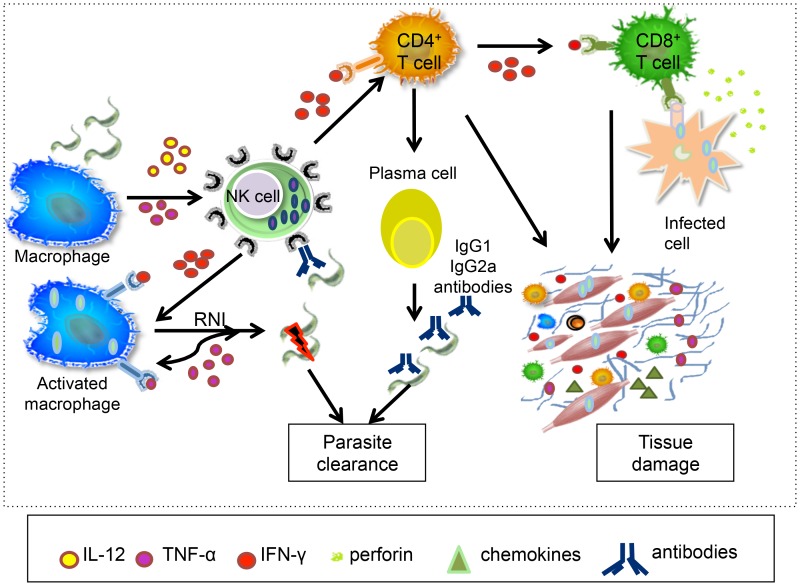
Immune response in the experimental murine model. Following infection with *T. cruzi*, the parasites infect and replicate in many nucleated cells. Innate immunity cells such as macrophages, dendritic cells, and NK cells provide the first line of defence against infection with *T. cruzi*, preceding the onset of the specific immune response by T and B lymphocytes. Parasite antigens induce macrophages to synthesize IL-12, a powerful inducer of IFN-gamma by NK cells. This inflammatory cytokine, together with TNF-alpha, triggers activation of macrophages and the inflammatory process, controlling parasite replication. Macrophage-derived reactive nitrogen intermediates (RNI) are directly associated with control of parasite burden. Differentiation and expansion of CD4+ and CD8+ T cells with polarization towards IFN- gamma are elicited by IL-12 derived from dendritic cells and NK cells, triggering cytotoxic activity by CD8+ T cells and effector mechanisms in macrophages. Effector CD4+ T cells stimulate B cells into proliferation and subsequent antibody production, which can lyse the trypomastigote forms. The acute phase is also characterized by recruitment of T cells to the tissues, in which IFN-gamma plays a major role by inducing chemokine production. In early immune responses, the inflammatory environment is crucial for host resistance to infection, but it might also lead to genesis of tissue damage. These immunological events were described in experimental models, and although the translation to human studies has limitations, they have elucidated many important aspects of *T. cruzi* infection.

The translation of animal findings to humans has limitations; however, the use of experimental models has elucidated many conceptually important aspects of the infection, highlighting the crucial role of inflammatory responses for establishment of immune-mediated mechanisms that drive disease outcome. These findings are summarized in Figure 4.

It is a consensus that during the acute phase, a robust immune response is mounted in the host, which leads to the dramatic control of parasitemia. Although the exact mechanisms that mediate parasite control have not yet been clarified in humans, it is believed that they rely greatly on the function of innate immune cells, such as natural killer (NK) cells, neutrophils, and macrophages. NK cells are important sources of interferon (IFN)-gamma and tumor necrosis factor (TNF)-alpha, which are critical for the activation of macrophages to eliminate the parasite. A phenotypic analysis of NK cells from children with acute Chagas disease showed a high frequency of a particular activated NK subpopulation, characterized as CD16+CD56− [8], which seems to be activated by parasite surface molecules [9]. Amongst these molecules, particular attention is given to glycosylphosphatidylinositol mucins (GPI-mucins), which are the most abundant *T. cruzi* surface molecules involved in parasite adherence to host tissues and critical in activating immune responses [10]. Thus, it is believed that proinflammatory cytokines released by macrophages and NK cells in response to GPI-mucins and other molecules further activate these and other cell types to control parasitemia [11].

While T cell activation is also observed during acute phase, the appearance of antigen-specific cytotoxic T cells is delayed, possibly due to an immunosuppression observed in acute patients [12]. Early activation of specific CD4+ T cells has been associated with a bias repertoire, showing a decreased frequency of CD4+ T cells expressing the T cell receptor V-beta region (Vbeta-TCR) 5 in the peripheral blood of acutely infected individuals from Bolivia, compared with uninfected individuals from the same geographical area [13]. Interestingly, chronic patients displayed an increase in the frequency of CD4+ T cells expressing the same Vbeta region [13], [14]. Recently, our group demonstrated that these Vbeta5 expressing CD4+ T cells display a highly conserved CDR3 region amongst cardiac, but not indeterminate patients [14].

The onset of adaptive immunity is followed by enhancement of circulating activated B cells [15]. After 15 days of infection, IgM antibodies are highly abundant in the sera of acute chagasic patients [16] and used as a serological parameter [17]. Still, in the early phase of infection, specific IgG and lytic antibodies against trypomastigotes are widely detected, which may be a mechanism of enhancing resistance to the parasite [16].

Generation of cellular and humoral immune responses to infection with *T. cruzi* is orchestrated in great part by cytokines. As demonstrated by studies with acutely infected children from an endemic area in South America, there is a dominant Th1 type (IFN-gamma) cytokine profile, with very low levels of IL-4 [18]. At this stage of infection, IFN-gamma may have a key role in controlling parasitemia, as mentioned before. In addition, there is evidence that IFN-gamma may act synergistically with benzonidazole during the acute phase, helping parasite clearance [19].

Most individuals that enter the chronic phase remain in the indeterminate clinical form, which is asymptomatic and represents an excellent example of equilibrium between the parasite and its host [20]. The asymptomatic, indeterminate form of Chagas disease has been associated with predominant production of regulatory cytokines such as IL-10, over inflammatory cytokines such as IFN-gamma and TNF-alpha [21], [22]. Thus, it is possible that the ability to produce IL-10 later in the acute phase will be important to control the response and allow for disease chronification. It is noteworthy that a fine balance between inflammatory and anti-inflammatory cytokines and an effective cellular response needs to be reached to keep parasite levels in check while avoiding tissue damage.

**Figure 3:**
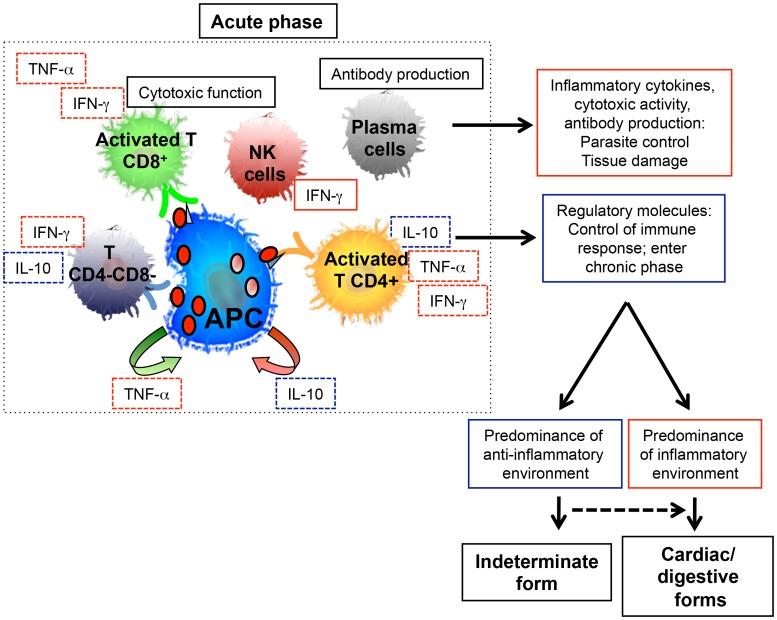
Clinical evolution of human Chagas disease. During the acute phase of human Chagas disease, macrophage and NK cell activation occur, as well as antibody production by plasma cells. These events lead to control of the parasite levels, observed in the late stages of the acute phase and throughout the chronic phase. NK cells and macrophages produce cytokines that might activate other cells such as CD4+, CD8+, and other T cell subpopulations, although the participation of these cells during the acute phase is not clear. The dotted boxes around some cytokines indicate that the cells associated with them may be responsible for their production, however, there is yet to be a definitive study confirming this. The production of IL-10 and other anti-inflammatory molecules may influence control of the response, decreasing tissue damage and allowing for the disease to enter into the chronic phase. In other studies, there is much evidence supporting that the predominance of an inflammatory environment during the chronic phase is associated with symptomatic forms (cardiac and digestive), whereas the predominance of an anti-inflammatory environment is associated with the maintenance of the indeterminate form.

These studies have provided limited, yet important, information helping to define the immune response in acutely infected individuals that allows for the establishment of hypotheses explaining the influence these events have on chronic disease evolution (Figure 3). Further studies to clarify the mechanisms involved in the immune response mounted during the acute phase will greatly empower us to understand the influence of the early immunological events on the differential clinical evolution observed in the chronic phase and, hopefully, assist us in developing interventions designed to avoid pathology development. Importantly, studies that can characterize and compare the immune response of individuals acutely infected via different routes (natural, oral, congenital, transfusion, and transplantation) will be of great value.

## Acute Chagas Disease in Immunocompromised Patients: Importance for Transplantation and HIV Management

The peculiar epidemiology of Chagas disease, with different forms of transmission and parasite isolates, has faced a new challenge: the reactivation of Chagas disease in patients with impaired cellular immunity. At least two groups of individuals are susceptible to Chagas reactivation due to immunosuppression: HIV co- infected individuals and transplant patients. Parasitemia is the most defining feature of reactivation [23], which usually presents with severe symptoms such as myocarditis [24] and meningoencephalitis [25].

In HIV-infected (or AIDS) patients, *T. cruzi* has emerged as an important opportunistic pathogen. It has been shown that between 20% and 40% of individuals co-infected with HIV and *T. cruzi* may experience disease reactivation, with parasitemia levels as high as the ones observed in the acute infection. It has also been observed that death is more common and occurs earlier in co-infected patients, as a result of complications related to both diseases. Meningoencephalitis is the most common manifestation associated with co-infection in individuals that experience disease reactivation, and the mortality rate may reach 100% [26]. Clinical studies have shown that reactivation of Chagas disease is directly associated with increased HIV levels and decreased CD4+ T cells counts in the peripheral blood [27]. Interestingly, patients who do not present reactivation of Chagas, but are co-infected, display higher CD4 counts and produce higher levels of IFN-gamma, TNF-alpha, and especially IL-4, than the HIV single-infected individuals [28].

Once HIV is diagnosed, *T. cruzi* specific treatment should be taken into consideration prior to the event of severe immunosuppression in order to either prevent or minimize the risk of Chagas disease reactivation. Although the duration of the treatment for immunocompromised patients has not been standardized yet, it is recommended the treatment should be continuous during immunosuppression, as it is for organ transplantation cases. Adverse side effects are frequent and there is a limited efficacy in achieving parasitological cure [29].

Another important situation in which patients may experience Chagas disease reactivation is due to immunosuppression previous to transplantation. This may occur inadvertently, in cases in which the patient undergoes transplantation without knowing that he/she has Chagas disease (usually in asymptomatic individuals in non-endemic countries), or due to heart transplant as a result of Chagas-related cardiac damage. Recent studies have shown that recurrence of Chagas disease may also occur in patients with lymphoreticular neoplasias, especially acute lymphocytic leukemia or Hodgkin's disease [30]. One challenge of identifying Chagas disease in immunosuppressed patients is that sera conversion may not occur. Thus, it is important to use other methods, such as PCR, to identify the parasite, allowing for early therapeutic intervention. It is recommended that exams be performed post-transplantation in intervals of one or two weeks for up to six months.

Patients with advanced Chagas cardiac disease may be eligible for heart transplant as a clinical management strategy for dilated myocardiopathy [31]. In such cases, also as a result of immunosupression prior to the transplant, parasite levels may increase and disease reoccur. When a patient is known to have Chagas disease, specific treatment is immediately introduced and the results are usually successful. Another consideration is with regards to the use of organs from Chagas patients to healthy recipients. Transplantation of the kidney or liver from an infected donor resulted in transmission in 19% and 29%, respectively [32], while transplantation of the heart from a *T. cruzi*–infected donor is contraindicated.

## Concluding Remarks

The epidemiological data reviewed in this article demonstrate that despite the successful initiatives to eliminate *T. cruzi* transmission by *Triatoma infestans* in certain endemic countries of Latin America, the coexistence of *T. cruzi*, their reservoirs, and vectors persists as a health problem, leading to outbreaks caused by oral and vectorial transmission. Migratory globalization has brought Chagas disease to a new global scenario. Important challenges remain associated with this picture, as discussed, such as prevention and control, early diagnosis, and therapeutic intervention.

Understanding how the immune response mounted in the acute phase can interfere with the development of pathology in the chronic phase is critical. To this end, identifying antigens that lead to immune activation early in disease and that might direct the response towards a protective one, is a fundamental discovery that remains to be achieved. Another level of complexity is added by the fact that the maintenance of health requires a fine balance between an activated response that leads to parasite control and a regulatory response that avoids tissue damage. Integrated approaches that combine studies of parasite and host factors, from clinical, genetic, and molecular stand-points will certainly be essential to understand the effects of the acute phase in disease development. Moreover, the discovery of cellular, genetic, and clinical biomarkers of disease progression and severity is a critical aspect to guide new medical practices towards prevention of pathology. Additionally, identification of biomarkers of treatment efficacy is another critical aspect in the management of Chagas patients. Identifying reliable biomarkers is, thus, an urgent need in Chagas disease. Cooperative efforts amongst scientists of different expertise and access to modern technology are, more than ever, necessary to battle Chagas disease.

## Methods for Search Strategy and Selection Criteria

Data for this review were identified by searches of MEDLINE, PubMed, and references from relevant articles using the search terms: “acute Chagas disease AND outbreaks,” “epidemiology of Chagas disease AND new cases,” “Chagas disease AND Europe,” “Chagas diseases outbreaks AND oral transmission,” “Chagas disease AND non-endemic,” “acute Chagas disease AND Amazon,” “oral infection AND Chagas,” “oral Chagas disease AND Latin America,” “Transplantation AND *T. cruzi* infection,” “Casos agudos de chagas no Brasil,” “Chagas disease AND AIDS,” “Reactivation AND Chagas disease,” “Chagas disease AND immunesupression.” Abstracts from meetings were not included in the references, which concentrated on the indexed literature.

**Key Learning Points**

- How do acute phase events influence disease development?
- How do immunosuppressant conditions influence the course of infection?
- What are the key immunoregulatory events in acute phase, and how may their progression influence the chronic phase?

**Top Five Papers**

1. Nóbrega AA, Garcia MH, Tatto E, Obara MT, Costa E, et al. (2009) Oral transmission of Chagas disease by consumption of acai palm fruit, Brazil. Emerg Infect Dis 15: 653–655.
2. Antas PRZ, Medrano-Mercado N, Torrico F, Ugarte-Fernadez R, Gómez F, et al. (1999) Early, intermediate, and late acute stages in Chagas' disease: a study combining anti-galactose IgG, specific serodiagnosis, and polymerase chain reaction analysis. Am J Trop Med Hyg 61: 308–314.
3. Dutra WO, Menezes CA, Villani FN, da Costa GC, da Silveira AB, et al. (2009) Cellular and genetic mechanisms involved in the generation of protective and pathogenic immune responses in human Chagas disease. Mem Inst Owaldo Cruz 104: 208–218.
4. Ferreira MS, Borges AS (2002) Some aspects of protozoan infections in immunocompromised patients- a review. Mem Inst Oswaldo Cruz 97: 443–457.
5. Bern C (2012) Chagas disease in the immunosuppressed host. Curr Opin Infect Dis 25: 450–457.
